# A Pseudo-Infarction Pattern in the Setting of Esophageal Malignancy

**DOI:** 10.7759/cureus.12193

**Published:** 2020-12-20

**Authors:** Naji Maaliki, Jorge Verdecia, Ryan Nelson, Win Aung

**Affiliations:** 1 Internal Medicine, University of Florida College of Medicine – Jacksonville, Jacksonville, USA

**Keywords:** pseudo-infarct pattern, esophageal malignancy, electrocardiogram (ecg/ekg), septal myocardial, left anterior descending artery, qrs axis

## Abstract

A 57-year-old African American male was admitted for workup of unintentional weight loss. He was found to have an esophageal squamous cell carcinoma. Electrocardiogram (ECG) readings demonstrated deep Q waves in leads V1-V2 with T wave flattening, which raised concern for a septal infarct. Myocardial infarction (MI) was subsequently ruled out through clinical, imaging, and laboratory analysis. The ECG findings were deemed as a pseudo-infarct pattern in the setting of squamous cell carcinoma of the esophagus.

## Introduction

Cardiac clearance is often required before surgery to stratify high-risk patients and avoid complications. Our focus is on the presence of ischemic heart disease using electrocardiogram (ECG) interpretation. Deep Q waves on ECG are usually associated with myocardial scarring, commonly from a myocardial infarction (MI) [1, 2]. Their presence can raise concern, especially in patients in dire need of time-sensitive surgery. We present a case with septal Q waves ascribed to a newly diagnosed esophageal malignancy, thus labeling it as a pseudo-infarct pattern. Determining the cause of the Q waves is essential to patient outcomes, as it can avoid unnecessary interventions and potential delays in care.

## Case presentation

**Figure 1:**
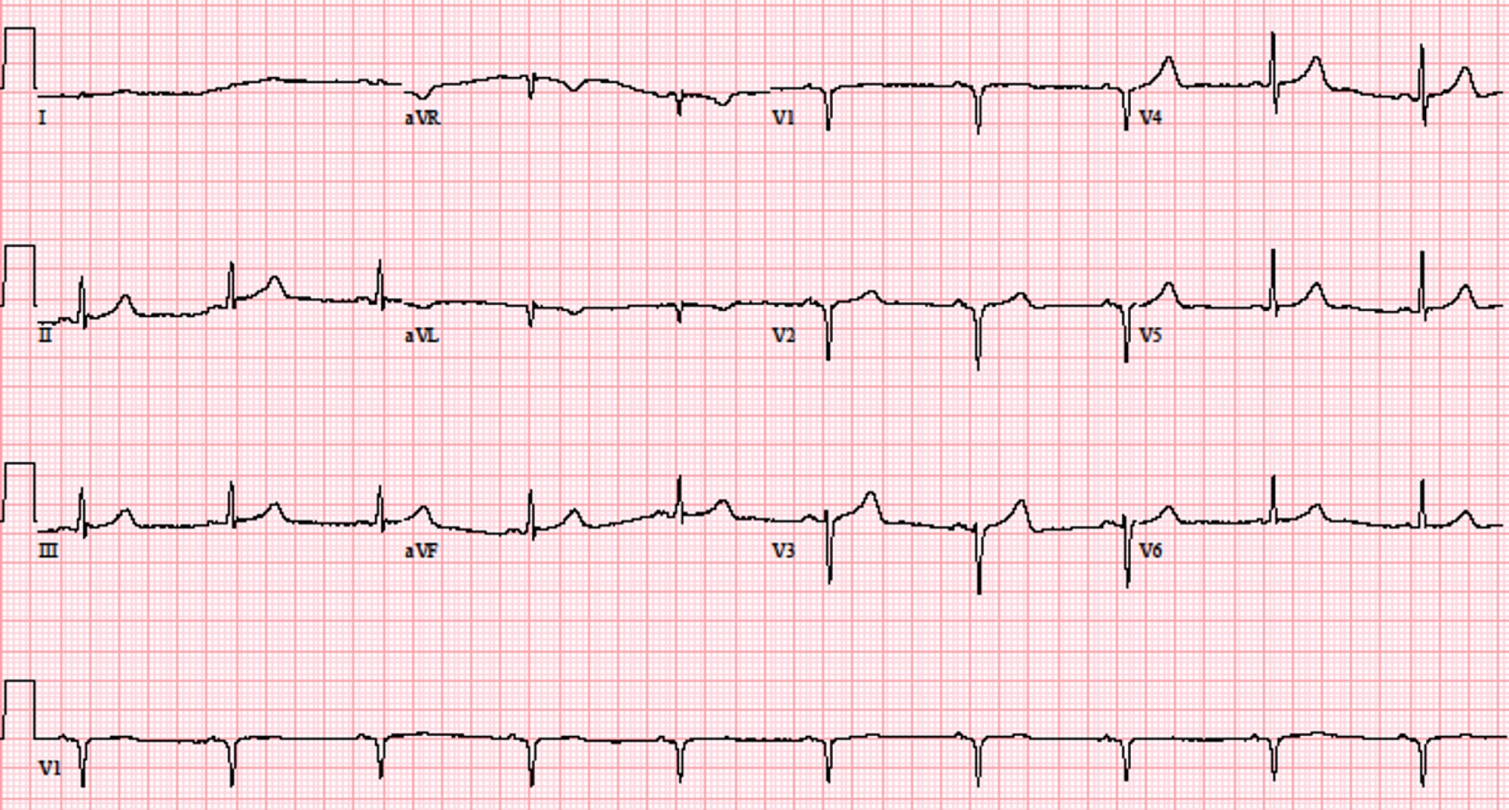
Electrocardiogram at presentation displaying deep Q waves in V1-V2 indicative of possible septal infarction

A 57-year-old African American male presented to our hospital with dysphagia and weight loss. His medical history was significant for tobacco and alcohol abuse. He had no prior cardiac history. Physical examination revealed cachectic appearance, epigastric abdominal tenderness, and cervical lymphadenopathy. Computerized tomography of the abdomen and pelvis displayed a distal esophageal mass. Esophagogastroduodenoscopy with biopsy revealed an esophageal squamous cell carcinoma. A subsequent positron emission tomography (PET) scan did not show distant metastasis. ECG revealed deep Q waves in leads V1-V2 with T wave flattening (Figure 1), which were concerning for a septal infarct pattern. The patient was hemodynamically stable and in no acute distress. He denied current or prior cardiac symptoms, including chest pain, dyspnea, palpitations, paroxysmal nocturnal dyspnea, orthopnea, or lower extremity swelling. Cardiology was consulted for cardiac clearance for planned esophagectomy. He received two points on the Revised Cardiac Risk Index; high-risk surgery (intrathoracic: esophagectomy) and the Q waves, making him a class III risk. Cardiac biomarkers were negative, and a bedside cardiac ultrasound revealed no wall motion abnormalities. An active or prior myocardial infarction was therefore deemed unlikely. The patient was then planned for an esophagectomy once he received neoadjuvant chemotherapy and radiation therapy. A port-catheter and a percutaneous endoscopic jejunostomy tube were inserted while he was inpatient, and a surgical oncology follow-up appointment was placed on discharge. 

## Discussion

A septal infarct can be determined from leads V1 and V2, with ischemic changes like pathologic deep Q waves with a deflection greater than 0.1mV and longer than 0.04 seconds, along with ST-segment and T wave alterations [3]. These findings raise concern, and in high-risk surgical situations, it is important to distinguish patients with significant coronary artery disease from those with a pseudo-infarct pattern. Noninfarct Q waves may be seen in a variety of cases such as anatomic variants, interventricular conduction abnormalities, myocardial injury, and lead misplacement [4]. Making this distinction is critical, as it can prevent further testing, which could delay life-saving procedures in some patients.

In our case, the patient developed deep Q waves in leads V1-V2 (Figure 1). While this was concerning for a septal infarct, acute MI was unlikely due to the absence of symptoms, negative serial cardiac biomarkers, and no regional wall motion abnormalities on transthoracic echocardiography. Previous MI was ruled unlikely due to a negative history of acute coronary syndrome (ACS) or angina equivalents and the aforementioned transthoracic echocardiography findings. Demand ischemia was also considered improbable due to consistent hemodynamic stability and normal perfusion indices. The ECG was repeated multiple times, ensuring proper lead placement. Additionally, he had a body mass index of 18 and did not have chronic obstructive pulmonary disease or gynecomastia, and further evaluation revealed a normal comprehensive metabolic panel and a negative autoimmune workup.

**Figure 2:**
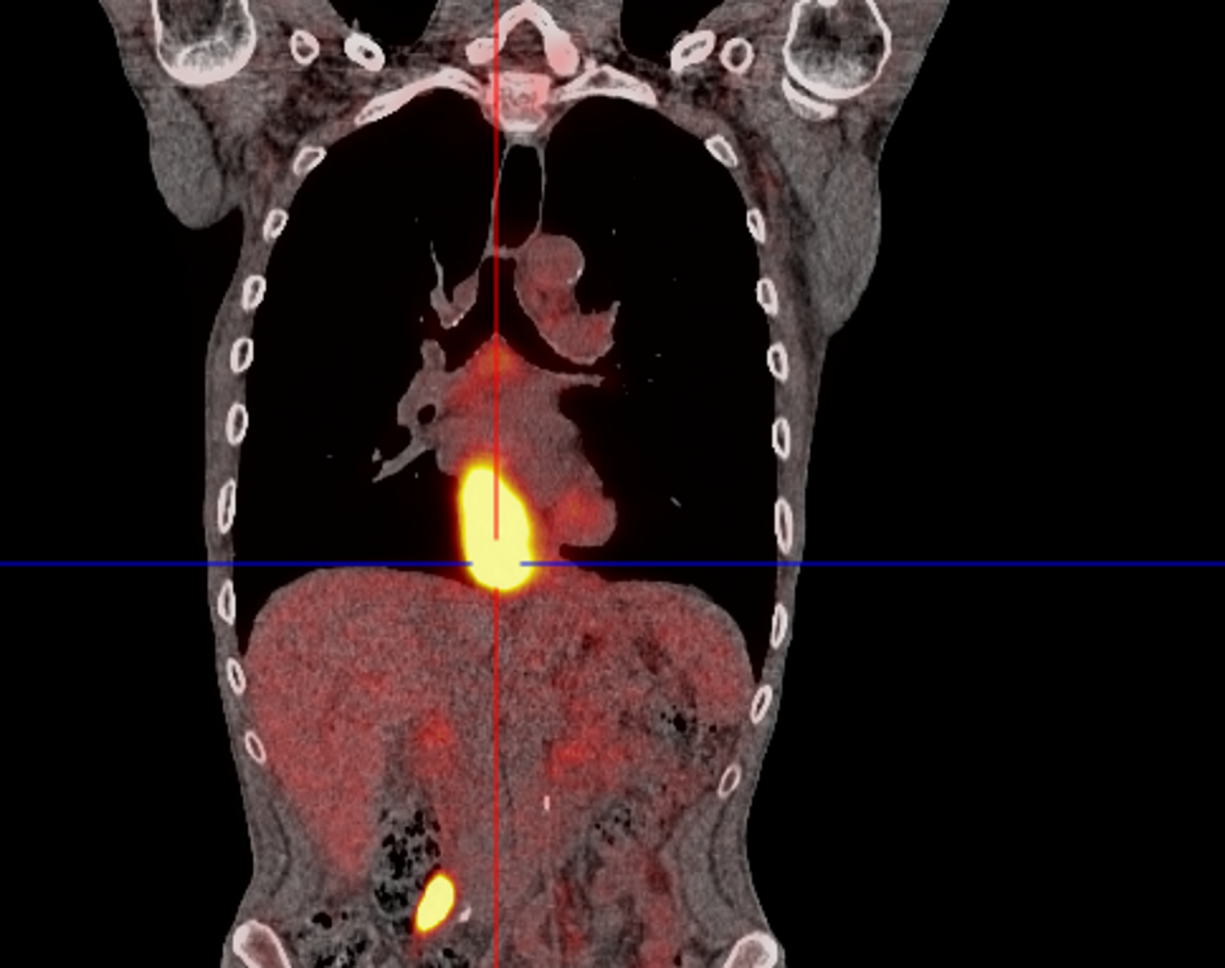
Positron emission tomography scan demonstrating esophageal mass located next to the heart

The recently diagnosed squamous cell carcinoma of the esophagus was attributed to being the pseudo-infarct pattern's potential cause. Although coronary angiography to definitively exclude vascular disease was not performed, the combined clinical and available investigative methods supported this notion. A pathological intrathoracic process, such as a neoplastic mass or increased pressure, may have shifted the heart's position relative to the ECG leads, thus altering the QRS axis and affecting the ECG reading [5-7]. Increased thoracic pressure may also interfere with the amount and type of matter between the heart and the chest wall [6]. Other mechanisms can be due to direct stretch and irritation of the cardiac myocytes and electrolyte abnormalities caused by a nearby hypermetabolic, damaged tissue [8]. The location and nature of this esophageal malignancy make the correlation possible (Figure 2). The lesion seemed to impinge on the heart's inferior surface, possibly causing the septal wall to shift. The hypermetabolic nature of the mass may have also led to the adjacent myocyte changes. 

## Conclusions

Esophageal malignancy can present with a pseudo-infarct pattern on ECG in the absence of significant coronary artery disease. The etiology may be multifactorial and has been associated with other malignancies and systemic diseases. Differentiating patients with a pseudo-infarct pattern from proper septal MI is essential, as failure to identify pseudo-infarct pattern may result in unneeded invasive tests and a delay in necessary interventions. In our case, this was significant as the patient needed to undergo a high risk, time-sensitive thoracic surgery.
